# Post-tuberculosis lung disease: Addressing the policy gap

**DOI:** 10.1371/journal.pgph.0003560

**Published:** 2024-09-05

**Authors:** Chase Yarbrough, Michael Miller, Mosala Zulu, Danielle Sharp, Afom T. Andom, Melino Ndayizigiye, Kwonjune Justin Seung, Paul Sonenthal

**Affiliations:** 1 Harvard Medical School, Boston, Massachusetts, United States of America; 2 Division of Global Health Equity, Brigham and Women’s Hospital, Boston, Massachusetts, United States of America; 3 Partners In Health–Lesotho, Maseru, Lesotho; 4 Department of Medicine, Brigham and Women’s Hospital, Boston, Massachusetts, United States of America; 5 Division of Pulmonary and Critical Care Medicine, Brigham and Women’s Hospital, Boston, Massachusetts, United States of America; University of Waterloo School of Public Health and Health Systems, CANADA

## Abstract

The burden of long-term functional impairment following curative treatment for tuberculosis (TB) constitutes a significant global health problem. By some estimates, chronic respiratory impairment, or post-tuberculosis lung disease (PTLD), is present in just over half of all patients who have completed TB therapy. Despite this high prevalence and substantial associated morbidity, discussion of PTLD is essentially absent from international and national TB policies and guidelines. Clear and ambitious clinical standards should be established for the diagnosis and management of PTLD, including the stipulation that all patients completing TB therapy should be screened for PTLD. Patients diagnosed with PTLD should receive linkage to chronic care, with access to inhalers and home oxygen, as indicated based on individual symptoms and pathophysiology. Leveraging their considerable influence, major funders, such as The Global Fund, could help close the gap in PTLD care by including PTLD in their strategic vision and funding streams. Immediate action is needed to address the substantial burden of disease associated with PTLD. This will require expanding the global approach to TB to include a commitment to diagnosing and treating long-term complications following initial curative therapy.

## Introduction

Sequelae from tuberculosis (TB) infection account for a substantial proportion of the overall morbidity associated with tuberculosis, contributing up to 47% of the disease burden in terms of Disability-Adjusted Life Years (DALYs) [1]. The disease can take a physical, economic, and social toll on its patients, who are frequently already vulnerable. Providing both medical and social support is necessary for appropriate care.

Post-TB lung disease (PTLD) is an increasingly recognized complication of tuberculosis, [2–5] The First International Post-Tuberculosis Symposium (2019) established the minimum case definition for PTLD as “evidence of chronic respiratory abnormality, with or without symptoms, attributable at least in part to previous tuberculosis” [6].

Despite increasing recognitioeeeeeeeeen of the problem among clinicians and academics, screening for the disease after curative therapy rarely happens in practice. The term “post-tuberculosis lung disease” is not included in the recently launched Global Tuberculosis Dictionary [7] and international organizations provide little, if any, guidance. The WHO consolidated TB guidelines contain no mention of PTLD [8]. At the national level, clear strategies for addressing PTLD are absent from National Tuberculosis Programs (NTPs), even in the countries with the highest burden of TB.

What are clinicians on the front-line to do? PTLD’s prevalence and morbidity demand a more prominent place in international guidelines and national policy.

### Patient vignette–Matlosa

Matlosa is a 35-year-old man from Maseru, Lesotho who presented to a dedicated multi-drug resistant tuberculosis (MDR-TB) treatment hospital with shortness of breath and lower extremity swelling. He was several months into his treatment for MDR-TB.

On presentation, he was in acute respiratory distress, requiring significant amounts of supplemental oxygen to maintain a normal oxygen saturation, and he was found to have acute decompensated right-sided heart failure—a dreaded complication from the chronic damage TB causes to the lungs.

At the hospital, he was continued on anti-TB therapy and given medication to stabilize his heart failure. He improved clinically and finished his treatment for MDR-TB while in the hospital. However, three months into his hospital stay, he remained dependent on supplemental oxygen to maintain his breathing. His dependence on oxygen was the only barrier to his discharge from the hospital.

Despite being the highest prevalence TB country in the world [9], resources to support patients with home oxygen are not readily available in Lesotho. Oxygen concentrators are in short supply, and many homes lack reliable electricity needed to run them. Despite these barriers, the non-profit organization Partners In Health-Lesotho (PIH-L) was able to arrange home oxygen for Matlosa, providing him with a concentrator, tubing, and back-up oxygen cylinders. PIH-L engaged contractors and electricians to ensure he had reliable home electricity and coordinated the recurring delivery of cylinder refills to his home.

Three months after discharge, he was doing well at home. He was still dependent on supplemental oxygen. He had restarted his fruit stand business and was preparing to get married.

## Epidemiology

PTLD is a heterogenous entity, encompassing any chronic respiratory abnormality attributable in whole or part to previous tuberculosis. Most PTLD prevalence estimates rely on spirometric data. The reported prevalence of impairment on spirometry after curative TB therapy varies considerably with estimates ranging between 28 and 87% [10]. A recent meta-analysis [5] that analyzed data from 41,014 patients estimated that 59.1% of patients had abnormal spirometry after treatment of tuberculosis compared to 5.4% of controls, although the assessed quality of the evidence was low and the heterogeneity in study outcomes was high.

Risk factors for development of PTLD include MDR-TB and multiple prior courses of TB treatment. Severity of airflow obstruction on spirometry increases proportionally to the number of times a patient has been treated for TB [11], and patients with MDR-TB have a lower percent predicted FEV1 after treatment compared to drug-sensitive TB [5].

It has been estimated that there are 155 million living TB survivors [12]. Assuming a PTLD prevalence of 59.1% [5], the overall global prevalence of PTLD would be approximately 91.6 million people. If this estimate is even close to accurate, the sheer number of people currently living with PTLD is startling and stands in stark contrast to the limited attention that PTLD receives in guidelines and policy.

### Existing guidelines and policies

The WHO consolidated guidelines on tuberculosis provide extensive, evidence-based recommendations for screening and treatment of tuberculosis, over the course of five modules and over 500 pages [8]. However, we were unable to find any mention of PTLD within its contents. Given the burden of disease, PTLD’s omission is notable.

We note similar omissions in national guidelines. India had the highest total number of new TB cases in 2022 [13]. India’s National Strategic Plan to End Tuberculosis provides a detailed strategy for the country’s TB strategy for 2020–2025 [14]. There is no explicit mention of PTLD, but there is a mention of the goal to “[a]ssess disability at the end of treatment and link those identified with disabilities to rehabilitation schemes (provided by government and/or others).” No additional details regarding screening or treatment are provided.

Lesotho is the country with the highest incidence rate of TB in the world [9]. Lesotho’s Tuberculosis National Strategic Plan 2023–2028 does not mention PTLD specifically, but post-TB care is at least materially discussed [15]. The plan mentions that there are “no guidelines on post-TB care and management and support” and recommends that post-TB care guidelines should be developed, but no specific recommendations for screening or treatment are provided.

National strategic plans (NSPs) for TB, in addition to the self-evident purpose of setting goals for a country, are also a key element of applying for funding from The Global Fund to Fight AIDS, Tuberculosis and Malaria (The Global Fund). The Global Fund approves resource allocation to a country ‘s funding request contingent on submission and alignment of NSPs. Because The Global Fund refers to the NSPs in considering funding allocations, the priorities of the funder play an outsize role in the interventions recommended in the NSPs. With that in mind it is notable that The Global Fund Strategy (2023–2028) makes no mention of PTLD. In the TB section (which is titled “End TB”), the focus is on “finding and treating all people with TB” and tuberculosis preventative treatment (TPT) [16]. These are noble goals, but it is possible (and one could argue, should be expected) to treat both TB patients and patients with PTLD. Furthermore, even if all cases of TB in the world at treated, many of those patients will still have PTLD at the end of therapy.

Although international organizations and national structures provide limited PTLD guidance, there are published clinical standards for screening and treatment. In 2021, Migliori et al. published a set of clinical standards based on a Delphi process [4]. The authors proposed the following six clinical standards:

1. Evaluate all patients at the end of TB treatment for PTLD
2. Evaluate patients with PTLD for pulmonary rehabilitation (PR)
3. Tailor the PR program to individual patient needs and the local setting
4. Evaluate the effectiveness of PR for PTLD
5. Conduct education and counseling for all patients receiving PR for PTLD
6. Address public health aspects of PTLD

These standards place a heavy emphasis on PR. This approach makes sense as PTLD is a heterogenous disease with varied findings on pulmonary function testing, and PR is the intervention with the highest likelihood of benefit across various types of PTLD. In 2023 Nightingale et al. advocated that the management of post-TB care should extend beyond PR and recommended medical management based on various clinical phenotypes of PTLD. However, they acknowledge that PTLD-specific data is lacking and therefore treatment for these phenotypes must currently be extrapolated from other contexts, representing an additional key knowledge gap [3].

### Current gaps

#### Diagnosis

International guidelines and national policies provide little to no guidance on diagnosing PTLD. Spirometry is the most commonly used diagnostic tool for assessing respiratory impairment after treatment of tuberculosis. While spirometry is useful for identifying obstructive and restrictive respiratory disease, it does not capture the full spectrum of PTLD. There are limited data on the use of other diagnostics such as non-spirometric pulmonary function tests (e.g. lung volumes or diffusion capacity), echocardiography, symptom-based scoring (e.g. the modified Medical Research Council scale [17] or the Health-related Quality of Life scale [18]), oxygen saturation, and 6 minute walk tests. These important measures are mostly confined to smaller studies [19–21].

Relying on spirometry to diagnose PTLD also misses other important associated morbidity, such as pulmonary hypertension. Pulmonary hypertension is suspected to be a significant source of post-TB morbidity, such as in the case of Matlosa presented above, but the burden of disease is unknown as it is rarely formally assessed [22]. Pulmonary hypertension from respiratory causes has a high mortality rate with a mean survival of 4.1 years [23]. While the gold standard diagnosis of right-heart catheterization is unlikely to be practical in many settings, diagnosis based on echocardiography provides reasonable test characteristics with a sensitivity and specificity of 83% and 72%, respectively [24].

The role of imaging in the assessment of PTLD is also an important consideration. Chest x-ray (CXR) and computer tomography (CT) of the lungs are the most commonly used imaging modalities in assessing tuberculosis and its sequelae. Abnormal imaging findings on CXR and CT after treatment of tuberculosis include cavitation, bronchiectasis, and fibrosis. The prevalence of these findings in PTLD patients is highly variable, [25] and the relationship between imaging findings and spirometric or functional impairment has not been well studied.

#### Linkage to care

Once PTLD is diagnosed, there is no standard guidance on how to link patients to follow-up care within the health system. After a diagnosis of PTLD is established, patients need to be linked to an outpatient system (such as a non-communicable disease [NCD] clinic) that can help them manage their illness and any associated comorbidities.

#### Treatment

International guidelines and national policies provide little guidance on treatment of PTLD. Current clinical standards provide recommendations for pulmonary rehabilitation [4] and limited recommendations on pharmacologic therapy [3]. Recommendations on treating chronic hypoxemia and pulmonary hypertension are lacking. Frontline physicians need the assistance of national policies and clinical guidelines to help them create a patient-centered treatment plan for each patient’s unique physiology.

#### Funding

Specific funding for the screening, diagnosis, and management of PTLD is limited, as is funding for research on PTLD. An online search of funding databases identified only one PTLD specific grant [26]. Major funders, such as the Global Fund and the United States Government provide support for specific diseases such as TB, HIV, and Malaria as well as non-specific funding for health systems strengthening, but fitting PTLD into those existing funding streams is a challenge.

### Recommendations

Given the substantial burden of PTLD, the lack of clinical and programmatic guidance, resources, and data are striking. PTLD should be considered an urgent global priority by funders, policymakers, and clinicians worldwide.

#### Guidelines and policies

Global and national guidelines and strategies, starting with the WHO consolidated guidelines on tuberculosis, should be updated to include PTLD. As a starting point, we propose consideration of the following objectives for incorporation into international guidelines and NSPs:

*1) Every patient who completes therapy for TB should be screened for PTLD*

Initial screening should take place at the conclusion of TB therapy and should include, at a minimum, spirometry, chest x-ray, clinical history, and a standardized symptomatic tool (e.g. mMRC or HRQOL). Additional diagnostic testing such as echocardiography, 6-minute walk test, and computed tomography can be considered based on individual clinical assessment. Screening for PTLD has the added benefit of improving a health system’s ability to diagnose other chronic respiratory diseases–as has been accomplished with the implementation of silicosis screening in Rwanda [27].

*2) Linkage to chronic care should be provided for all patients identified with PTLD*

Once patients are diagnosed with PTLD, they need to be linked to an outpatient system that can support the management of their chronic respiratory disease and any other comorbidities. TB treatment is sometimes provided under the auspices of national TB programs that operate specific TB clinics or “corners” within a health center, rather than a general outpatient department. Thus, after treatment for TB, patients need to be connected to another program, such as an NCD clinic that can continue their management. Improving PTLD care within an NCD program is an opportunity to strengthen NCD programs and NCD management more generally.

*3) Patients with PTLD should be provided pulmonary rehabilitation if indicated*

Pulmonary rehabilitation is an important cornerstone in the treatment of chronic respiratory disease, and thus it should be provided for PTLD patients who are likely to benefit, such as those who can tolerate some degree of exercise but whose health-related quality of life is affected by their respiratory impairment.

*4) Patients with PTLD should be considered for pharmacologic therapy tailored to individual clinical phenotypes*

The standard of care for PTLD should include an initial assessment of pulmonary function tests upon completion of tuberculosis treatment, followed by individualized therapy based on symptoms and pathophysiology. For example, patients with evidence of COPD would be treated according to standardized COPD guidelines [28], including inhalers such as bronchodilators and corticosteroids. However, the benefit of inhaled corticosteroids should be weighed against concerns about associated increased risk of reactivation of tuberculosis [29]. Inhaled trepostinil, although not currently widely available, has demonstrated improved exercise capacity in patients with pulmonary hypertension due to interstitial lung disease [30] and could be considered in PTLD patients with WHO Group III pulmonary hypertension.

*5) All patients with chronic hypoxemia should be provided with home oxygen*

In PTLD patients with chronic hypoxemia, treatment with long-term oxygen therapy should be provided. Limited evidence exists for benefit specifically in PTLD patients [31], but oxygen is the mainstay of treatment for hypoxemia and home oxygen has long been known to improve survival in a variety of conditions, including COPD [32].

Patients with PTLD and chronic hypoxemia must have access to home oxygen. The 76th World Health Assembly adopted a resolution calling for increasing access to medical oxygen [33]. However, the resolution focuses primarily on oxygen in health facilities, and it does not address access to home oxygen. Patients like Matlosa who are chronically dependent on oxygen must not be condemned to living in a healthcare facility indefinitely because of chronic hypoxemia. Clinicians must be equipped with the knowledge and resources to send patients home from the hospital with oxygen, if indicated.

National Oxygen Roadmaps supported by influential organizations, including the Global Oxygen Alliance (GO2AL), should include home oxygen for chronic hypoxemia as a key priority and advocate for its availability.

*6) PTLD-related outcomes should be included as essential endpoints in TB clinical trials*

Traditionally, clinical trials of new TB drug regimens have utilized microbiologic cure as the primary clinical endpoint. However, with the increasing recognition of the prevalence of chronic respiratory impairment after curative TB treatment, there has been a push to include post-TB endpoints in clinical trials [34]. New regimens should be evaluated not only by their success in obtaining microbiologic cure but also by their ability to prevent post-TB complications.

#### Funder leadership and prioritization

Major funders, like The Global Fund and the United Sates government, should leverage their considerable influence to establish PTLD as a priority. However, PTLD cannot be completely siloed within tuberculosis programming. As a chronic disease, addressing PTLD requires robust health systems that can provide long-term follow-up, ideally through linkage to established non-communicable disease (NCD) programs. Of course, it has increasingly been recognized that even vertical programs targeting diseases like TB also require strengthening healthcare systems more broadly in more of a “diagonal” approach [35] in order to effectively and sustainably treat the target diseases. In the most recent Global Fund strategy [16], there is an increased emphasis on providing funding for “resilient and sustainable systems for health” (RSSH), and The Global Fund now provides flexibility for countries to revise the funding split between disease programs and programs that provide RSSH. The Global Fund should specifically fund interventions for PTLD screening and treatment. For now, funding for these interventions should be provided under both the TB and RSSH funding streams. However, we need to combine our advocacy for the increasing recognition of the importance of PTLD with an acknowledgement of the shortcomings in international NCD funding and advocacy for improved NCD funding streams.

There will be an argument that the focus of policy dollars should be on the eradication of TB, and that eradication of TB will prevent all future cases of PTLD. Proponents of this view may claim that the research on PTLD is “immature” and highlight uncertainties about the best approaches to screening and treatment. This argument is misguided, and reminiscent of the old arguments that all HIV funding should be on prevention and not treatment. It is possible to tackle both active TB and PTLD at the same time. In fact, the substantial burden of disease posed by PTLD should make it imperative. The evangelists of “cost-effectiveness” would argue that it does not make sense to provide long-term oxygen support to patients like Matlosa. We posit that the patients experiencing these conditions would have opposing value judgements.

## Conclusion

Post-tuberculosis lung disease is a common and variably debilitating condition resulting from infection with tuberculosis, affecting tens of millions of people globally. Its significance is becoming more widely recognized. Despite increasing recognition of the burden of disease, international and national policy falls short on setting priorities for PTLD screening and treatment. WHO TB guidelines and individual national TB strategic plans need to be updated to provide guidance on the diagnosis and management of PTLD. Patients with PTLD should receive treatment based on their individual clinical phenotype, including access to home oxygen if indicated. Funding agencies, such as The Global Fund, should include PTLD in their funding priorities and strategic vision, and they need to fund work on PTLD screening and treatment. Without these shifts in policy and funding, other patients like Matlosa will be left without timely diagnosis and potentially life-changing treatment.
